# Repurposing cholesterol lowering drugs in the treatment and management of monkeypox

**DOI:** 10.1097/JS9.0000000000000010

**Published:** 2023-01-27

**Authors:** Saravanan Sekaran, Suresh Kumar R. Sekar

**Affiliations:** a Department of Prosthodontics, Saveetha Dental College and Hospitals, Saveetha Institute for Medical and Technical Sciences, Chennai, Tamil Nadu, India; b College of Natural Sciences, Arba Minch University, SNNPR, Ethiopia

*Dear Editor,*

While the likelihood of monkeypox (MPX) becoming a pandemic, very little is known about the virus. The smallpox-like symptoms caused by the MPX virus are less severe. Given the current possibility of a pandemic, it is crucial that any negative impacts of a MPX epidemic on public health be kept to a minimum. At the time of writing this letter, Centre for Disease control and Prevention has reported 77,092 confirmed cases across the globe in more than 89 countries. According to Centre for Disease control and Prevention, there is currently no cure for MPX infections; nevertheless, most patients recover after receiving supportive care, such as rehydration. MPX virus and Smallpox virus share genetic similarity and there is evidence to suggest that smallpox vaccination provides some protection against MPX, leading to a more favorable clinical presentation. To prevent high risk individuals, two vaccines namely JYNNEOS and ACAM2000 are used against MPX^1^. Among several pharmacological interventions suggested, antiviral drugs Brincidofovir and Tecovirimat show promising therapeutic effects. It inhibits the production of protein required for extracellular virus production^2^. Clinical trials of these antivirals are currently underway and may take a longtime to decipher its effectiveness and safety.

Learning from coronavirus disease-2019 (COVID-19) pandemic, several clinical trials and observational studies have shown that statins can protect against death and reduce the symptoms of respiratory infections^3^. In addition to this, cholesterol lowering drugs (fenofibrates and PCSK9 inhibitors) also presented beneficial effects in COVID-19 patients with hypercholesterolemia^4^,^5^. These drugs exhibit antiviral activity by altering cell membrane cholesterol and non–lipid-related pleiotropic effects such as anti-inflammatory and immunomodulatory effects^6^.

The entry of fusogenic, enveloped viruses into host cells requires lipid envelope, especially cholesterol. HSV and HIV-1 requires cholesterol on the host membrane and on the viral envelope for its entry. In envelope glycoprotein clustering of HIV-1, cholesterol is a major molecular platform regulating this process^7^. Cellular cholesterol depletion by statins reduced the production of human parainfluenza virus type 1 (hPIV1) particles by interfering virus assembly^8^. It also affects the stability, density of virions of influenza A virus and respiratory syncytial virus^9^. Lovastain and gemfibrozil inhibited hP1V1 virus assembly production through disruption of lipid raft integrity. Dengue virus infection induced the expression of proprotein convertase subtilisin/kexin type 9 (PCSK9), which decreases the recycling of low-density lipoprotein receptor to promote cholesterol redistribution into ER repressing ER-resident STING and type I interferon activation^10^. To combat this, PCSK9 inhibitors significantly improved the secretion of type I interferons. Statins are also repurposed in treating the patients following Ebola and Influenza infections. Overall it is clear that, statins exhibits antiviral effects by blocking/altering cholesterol trafficking, redistribution, virus attachment, virions production, maturation, release, and virions stability.

MPX seems to become another worldwide public health crisis, evoking parallels to the ongoing COVID-19 epidemic and its forebears. Repurposing cholesterol lowering drugs could be an effective approach in MPX. However, there is a lacune in understanding the role of cholesterol and its redistribution in MPX. Reducing cholesterol seems to be an effective antiviral approach, it could also dampen the host’s immune system^11^. One must also keep in mind that since viruses rely on lipid flux, employing statins to reduce cholesterol levels that is needed for viral maintenance rather than global reduction could be an effective approach. Hypercholesterolemic patients infected with MPX should continue taking cholesterol lowering drugs. Administering PCSK9 inhibitors, statins and fenofibrates could be considered as an adjuvant therapy along with standard drugs employed in in MPX treatment.

## Ethical approval

Not applicable.

## Sources of funding

None.

## Author contribution

S.S., S.K.R.S. performed literature search and drafted the letter.

## Conflicts of interest disclosure

The authors S.S. and S.K.R.S. certify that they have no affiliations with or involvement in any organization or entity with any financial interest (such as honoraria; educational grants; participation in speakers’ bureaus; membership, employment, consultancies, stock ownership, or other equity interest; and expert testimony or patent-licensing arrangements), or nonfinancial interest (such as personal or professional relationships, affiliations, knowledge or beliefs) in the subject matter or materials discussed in this manuscript.

## Research registration unique identifying number (UIN)

None.

## Guarantor

None.
